# Welshers

**Published:** 1903-10-15

**Authors:** H. McManus


### "WELSHERS."

"Welshers," as you no doubt know, is an idiom of the turf to describe one who accepts certain obligations without any intention of fulfilling them. And for purpose of consideration, we will divide the subject into four classes that appear in dentistry.

*First* is the man who welshes on his college. He comes here, or some place else, obtains an education, graduates, receives the right to prefix "Doctor" to his name, and goes out into the world. In how *many cases* does he remember the institution that made him possible? In how *many cases* does he remember the men who taught him which side of an impression to pour? In how *many cases* does he regard his college as anything but a place he once believed the best on earth, but now wonders how he thought so?

If he prospers, he attributes it to his own personal qualities, and feels that his college should take pride in him. That his old professors should meet him hat in hand, and if they don't—and to their credit and good sense be it said they rarely have—he becomes in his own mind a much-abused but superior person, too good to associate with even the man who taught him all he knows. For this welsher seldom learns anything after he has once joined the band.

He has forgotten *every* obligation his diploma imposed upon him, and he has welshed on every promise he ever made.

*Second* comes his brother, who welshes on his society. He comes home from college flushed with pride, and ambitious for success. He joins his State and local societies, for he is willing to associate himself with men of his calling; incidentally, let me say that the dental societies of this country are the greatest post-graduate schools of the profession. He attends his first meeting, and like as not sees that the local paper makes mention of the fact. But after a few years he loses interest, and becomes one of the little band that stand around the outside door, smoking bum cigars, and finding fault with everything, from the ventilation of the hall to the color of the essayist's necktie, and talking among themselves of how much better they could run things.

Does he ever offer to write a paper, or give a clinic? Does he ever volunteer to help the executive committee that he abuses get the hall ready for the exhibitors? Not he. As a mark of particular consideration, sometimes he will agree to open a discussion, if he is offered as an inducement the principal paper of the day. But when the time comes, first, he will accuse the secretary for not notifying him, or throw the blame on the post-office department for not receiving an advanced copy. And then pitch into the lecturer on general principles, as if he were his born enemy, and disagree with everything he cannot understand. At the end he goes home so pleased with his own success that the treasurer will never succeed in collecting his dues.

Where are the promises he made when he signed the constitution? Where are the great things he told the "doorstep gang" he could do? All welshed out on.

*Third*, we have the cousin of these, who, having forgotten his college, and forsaken his society, makes up his mind "to get all there is out of the business." And so he goes to work with but one end in view, and that one end is himself. You ask him of society matters. He tells you, with an air of frank and honest affrontery, that he doesn't know, that he hasn't time to waste on such trash, that when he gets a day off he goes fishing. You ask him of his college. He says "it's too far away, he hasn't been there in years;" "they don't run the old shop as it should be, nowadays anyway." He thought he would go there last winter when the commandery met in the old town, "but he got in with the boys, and they were 'such a hot bunch of babies he couldn't break away.'"

He earns his living pounding gold and scraping rubber, but as for professional feeling he might as well be a door-mat. Of the three he is the worst, because, nine times out of ten, he is a good fellow, and a clever mechanic, but for all the good he does dentistry he were better in the moon. He has welshed on his profession.

*Fourth* and last is the hypocritical welsher. He always attends the commencement exercises, and sits well forward on the stage, but never by chance even comes to the Alumni meeting. For the same reason he joins every dental society for miles around, but never can he stay through a session. Ask him to read a paper? "Why, of course." His reply delights your soul. Ask him to give a clinic? He "would be delighted to." And while his name appears on the program, when the time arrives *he* does *not* appear. Sometimes he writes a most polite note. "So sorry, but an old patient required his services—next year without fail."

He's an amiable, sociable sort of a chap, and as he's amusing, you rather like him. He tells you how pretty his office is furnished, and how his friends think it a pity he is a dentist, because he is so clever with his brush, and his pen, and his pencil. And how with his sympathetic temperament what a fine practice he could have had as a physician if he had only started right. Sometimes in a burst of confidence he'll tell you that he intends to "shake the dirty business some day," but in the meantime he goes his way putting in fillings you can pick out with cotton pliers, and setting bridges you can take off with your fingers.—buncoing the public out of good money, and leaving a trail of dead teeth in his path. Fortunately there are not many of him, but one to a town is one too many, for he is welshed on himself.

These are examples of the worst. And you will notice I have said throughout he—for the women of our profession are always earnest, serious and thoughtful.

Of the best, you all know. The sincere, hard-working, honest gentlemen, whose efforts have made our calling an honor to the country, and whose names will go down to posterity as benefactors to mankind. So much has been said of the good that I, being naturally perverse, have tried to picture the bad as he exists in every community. Not to gloat on his iniquity, but to ask your help in an effort to reform, for reform is so easy that it is within reach of all.—Dr. H. McManus, in *Stomatologist.*

